# HIV-HBV Coinfection—Current Challenges for Virologic Monitoring

**DOI:** 10.3390/biomedicines11051306

**Published:** 2023-04-28

**Authors:** Simona Ruta, Laura Grecu, Diana Iacob, Costin Cernescu, Camelia Sultana

**Affiliations:** 1 Virology Discipline, “Carol Davila” University of Medicine and Pharmacy, 020021 Bucharest, Romania; madalina.sultana@umfcd.ro; 2 Department of Emerging Viral Diseases, “Stefan S. Nicolau” Institute of Virology, 030304 Bucharest, Romania; laurapolosanu@gmail.com; 3 Department for the Prevention and Control of Healthcare Associated Infections, Emergency University Hospital, 050098 Bucharest, Romania; dianagiacob@gmail.com; 4 Romanian Academy, 010071 Bucharest, Romania; cernescu@racai.ro

**Keywords:** HIV-HBV co-infection, public health, pathogenesis, biomarkers, RT-PCR, ddPCR, antiretroviral treatment

## Abstract

HIV-HBV coinfected patients have higher rates of liver-related morbidity, hospitalizations, and mortality compared to HBV or HIV mono-infected ones. Clinical studies have shown an accelerated progression of liver fibrosis and an increased incidence of HCC, resulting from the combined action of HBV replication, immune-mediated hepatocytolysis, and HIV-induced immunosuppression and immunosenescence. Antiviral therapy based on dually active antiretrovirals is highly efficient, but late initiation, global disparities in accessibility, suboptimal regimens, and adherence issues may limit its impact on the development of end-stage liver disease. In this paper, we review the mechanisms of liver injuries in HIV-HBV coinfected patients and the novel biomarkers that can be used for treatment monitoring in HIV-HBV coinfected persons: markers that assess viral suppression, markers for liver fibrosis evaluation, and predictors of oncogenesis.

## 1. Introduction

The World Health Organization’s sustainable development goals for 2030 aim to end the epidemic of AIDS, combat hepatitis and other communicable and sexually transmitted diseases by 2030 [1]. Coinfection with Human Immunodeficiency Virus (HIV) and hepatitis B virus (HBV) is a global public health problem, with a more severe outcome than HBV or HIV mono-infections, including an increased risk for liver-related morbidity and mortality [2]. The rates of significant clinical events, liver-related hospitalizations, as well as the incidence of hepatocellular carcinoma (HCC) are much higher in HIV-HBV coinfected patients than in HBV or HIV mono-infected ones, and liver mortality is still one of the leading causes of non-AIDS deaths in people living with HIV/AIDS (PLWH), including very young ones [2]. Various mathematical modeling studies using ordinary differential equation models, fractional order models, approaches based on modern evolutionary computational techniques, and Padé approximation have been used to improve the understanding of HIV and HBV viral dynamics and to predict clinical evolution and response to treatment [3,4,5].

Worldwide, it is estimated that 5–20% of PLWH are also chronically infected with HBV [6]. Extremely high prevalence rates of HBV coinfections in PLWH are reported in Central and Western Africa (median: 16.4%), as compared to very low prevalence rates reported in Eastern Asia (China: 0.4%) [7]. Both viruses have similar routes of transmission and can be acquired via parenteral, sexual, or materno-fetal (mainly perinatal) ways. It is estimated that 25% of PLWH can acquire HBV during adulthood (a pattern seen in areas with low HBV prevalence, such as the USA or Western Europe [8]), while 50–90% are co-infected at birth or in early childhood [2] (a pattern frequently reported in Africa and in countries from Eastern Europe before the introduction of universal immunization programs [2,6]).

Recent data report high global HIV-HBV coinfection rates in several vulnerable groups: people who inject drugs (11.8%), men having sex with men (6.1%), commercial sex workers, and heterosexual adults (6.1%) [7]. There are significant differences according to the geographic area [7,9,10,11,12,13,14,15,16,17], with spots of high prevalence (for men who have sex with men—Germany in Europe [18] or Taiwan in Asia [19], for commercial sex workers—Central African Republic in Africa [20]). Still, data are missing for many regions, mainly due to difficulties in reaching risk groups. The accessibility of harm reduction measures is an equally important determinant of HIV and HBV prevalence, and stigma remains an important obstacle in the development of effective policies for diagnosis, treatment, and prevention in vulnerable groups.

HIV-HBV coinfected patients must be treated for both infections using dually active antiretroviral drugs [21,22]. The first-line therapy for HIV-HBV coinfection is based on tenofovir, administered either as tenofovir disoproxil fumarate (TDF) or tenofovir alafenamide (TAF)—a prodrug of TDF which preserves its high antiviral potency and high barrier to resistance while leading to fewer renal and bone side effects [21]. Regimens based on lamivudine (3TC) or emtricitabine (FTC) monotherapy, once suggested as acceptable in resource-limited settings for patients with undetectable or low plasma HBV-DNA [23], are no longer recommended. Other regimens sparing 3TC/FTC/TDF or TAF must be avoided due to their risk of hepatitis reactivation.

Antiretroviral therapy (ART) has a major impact on the evolution of HIV-HBV coinfection, especially when initiated early in subjects without severe fibrosis, as it decreases both HBV and HIV viral loads and prevents flares of hepatitis reactivation due to immune reconstitution in patients with severe immunosuppression [24]. However, worldwide, only 16.7% of HBV mono-infected patients receive treatment [8], as access to dually active antiretrovirals has been limited until recently in low-income countries, where most at-risk populations remain untreated [25]. Although important progress has been made by scaling-up tenofovir-based ART for PLWH [7], important local and regional disparities in accessibility persist, and high proportions of coinfected patients had suboptimal treatments, which rapidly select resistant HBV strains, altering the long-term evolution of the liver disease.

These epidemiological and therapeutic data are concerning, and there is a continuous need for patient monitoring and testing. The aim of this review is to assess the diagnostic and therapeutic challenges in HIV-HBV coinfected persons and to bring a novel perspective on the current and future biomarkers used for virologic monitoring and treatment prioritization.

## 2. Pathogenesis of Liver Injuries in HIV-HBV Coinfected Patients

According to a large multicentric cohort study performed in the early 2000s, the rate of liver-related mortality was significantly higher in people with HIV-HBV coinfection, namely 14.2/1000 person-years, compared with 1.7/1000 person-years in HIV mono-infection and 0.8/1000 person-years in HBV mono-infection [8].

Although the rate of liver-induced deaths has decreased over time, liver fibrosis can still be observed in up to one-third of all cases, even in individuals who are undergoing long-term ART [26]. Additionally, the incidence rate of hepatocellular carcinoma in HIV-HBV coinfected patients can reach up to 2/1000 person-years [27,28]. Interestingly, the adherence to HCC screening protocols in HIV-HBV coinfected is significantly lower than in HIV-HCV coinfected patients and remains below 20% in all cases of HBV and/or HCV coinfection [29].

**Figure 1:**
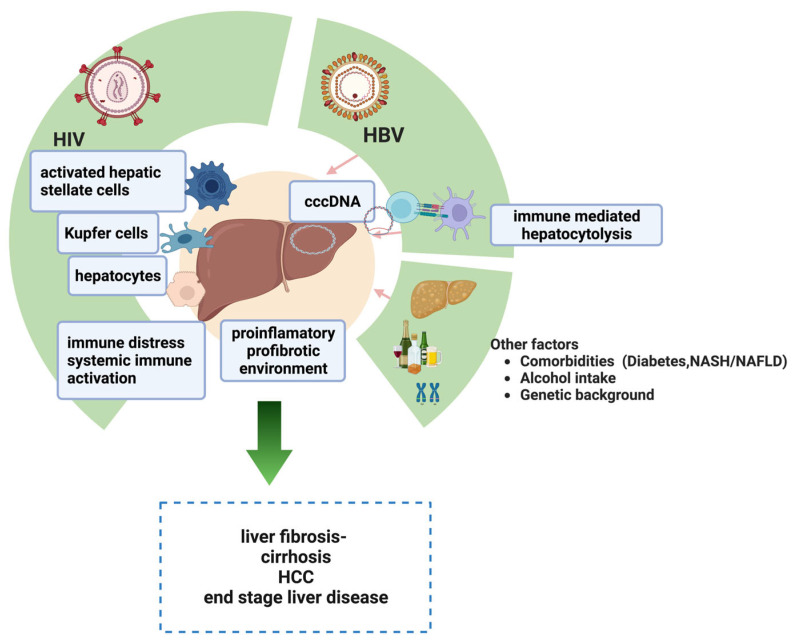
Mechanisms of liver injuries in HIV-HBV coinfected patients. The pathogenic mechanisms derive from the combined influence of HIV and HBV replication inside the liver, the associated immune distress and immune-mediated hepatocytolysis, and the additional host-related risk factors (comorbidities, genetic background, ART response). Created with BioRender.com (accessed on 26 April 2023), with a license to publish.

The specific pathogenic mechanisms of liver injuries in HIV-HBV coinfected patients include the combined action of HIV and HBV replication, immunosuppression, and host-related comorbidities or genetic factors (Figure 1) [2].

### 2.1. HIV-Related Factors

The progression of HBV-induced liver disease is accelerated by the coinfection with HIV [30]. HIV acts through multiple direct and indirect mechanisms to trigger or amplify liver injuries [17,31]. On the one hand, HIV can bind to CXCR4 receptors expressed on the hepatocytes and can persist in the liver even during ART treatment [32,33], destabilizing the cytokine environment and promoting inflammation [34,35], as well as liver fibrosis (already seen in 8–10% of HIV mono-infected patients) [36,37]. Moreover, HIV exerts both a direct and indirect effect on liver hepatic stellate cells [31], which are key players in both the hepatic immune responses, fibrogenesis, and possibly in carcinogenesis through transdifferentiation in- or regulation of- hepatic stem cells [38]. HIV enters hepatic stellate cells via a CD4-independent route and can be transferred through direct cell contact to intrahepatic lymphocytes, maintaining an active local replication. Viral antigens, such as gp120, can trigger or amplify a proinflammatory/profibrotic response of activated hepatic stellate cells [39]. In addition, HIV-infected T helper cells lose their ability to impede profibrotic signaling by NK cells [40,41]. An indirect effect of HIV infection on other cell populations, such as Kupffer cells, can further amplify liver injuries in coinfected patients [42,43]. These and other more specific mechanisms have also been involved in liver fibrosis development during HIV-HCV coinfection [44].

HIV- induced CD4 T lymphocyte depletion and systemic immune activation is followed by an inadequate innate and adaptive response, with the consecutive release of proinflammatory and profibrotic cytokines (TGF beta, IL-1, IL-6), increasing the risk of liver fibrosis. HIV-HBV coinfected patients with high HBV viral loads have slower rates of CD4+ T cell restoration under treatment [45] and maintain a higher risk of mortality even after early initiation of tenofovir-based therapy [46]. In addition, coinfected patients with severe immunosuppression and high HIV viral load can experience immune-mediated liver injuries, with hepatic flares, during the immune reconstitution syndrome, that can paradoxically develop 4–8 weeks after the initiation of antiretroviral treatment [47]. ART introduction may lead, in some cases, to the reactivation of chronic hepatitis or decompensation of liver cirrhosis [48].

### 2.2. HBV-Related Factors

Immunopathogenesis is the main mechanism of liver disease progression in chronic hepatitis B, where HBV intrahepatic replication can occur even in the absence of detectable viremia [49]. Exhausted or tolerant HBV-specific CD8+ T cells [50,51], as well as suppression of CD4+ T-cell responses, mediated by dendritic cell impairment [52] or by upregulation of PD-1 expression [53,54], and amplified by HIV-induced immunosuppression, can contribute to disease chronicization. HBV can also suppress TLR-mediated immune responses and promote TGF-β expression, therefore activating the proinflammatory cytokines and accentuating the cytokine imbalance produced by HIV infection, although this mechanism was not confirmed as a key player in liver disease progression [49,55]. Activation of hepatocyte Fas/FasL pathways following Hepatitis B surface antigen (HBsAg) production during HBV replication might also be important for liver disease progression [56].

### 2.3. Other Contributing Factors

Several comorbidities (especially diabetes, non-alcoholic fatty liver disease, non-alcoholic steatohepatitis), heavy alcohol use, and the baseline level of liver fibrosis contribute to the development of liver complications in HIV-HBV coinfected patients [31,32].

The prevalence of diabetes varies across different cohorts from the United States and Europe. Up to 10% of HIV-infected adults can develop diabetes versus up to 8.3% of the cases in the general population [57]. However, the risk of diabetes in adults with HBV or HCV coinfection remains unclear. Data from a large Canadian cohort showed a lower prevalence in coinfected subjects (8.1% in those HIV-HBV coinfected and 4.9% in HIV-HCV co-infected [57]).

Data on alcohol use and alcoholic liver disease in PLH is limited. Approximately 16–22% of individuals with HIV infection report an increased average of current or lifetime alcohol consumption vary [58]. According to Butt et al., the rate of alcohol use is higher in HIV-HCV coinfected individuals than in HIV-HBV coinfected ones (45% vs. 16.2%), but the rate of alcoholic liver disease in the latter population is unclear [59].

The rate of non-alcoholic fatty liver disease (NAFLD) in HIV mono-infected adults varies between 9 and 73%, while the prevalence in HIV non-infected adults reaches up to 25% [60]. Several studies indicate high percentages of NAFLD in HIV-HBV coinfected patients, varying from 15 to 73% on liver biopsies [61]. HIV-HBV coinfected patients with non-alcoholic steatohepatitis (NASH) display higher progression rates for both fibrosis progression, cirrhosis, and hepatocellular carcinoma compared with HIV mono-infection. This is probably due to various HBV-linked molecular mechanisms that modulate the expression of cholesterol synthesis genes and dysregulate the cellular signal transduction pathways affecting cell growth and apoptosis [62,63].

The influence of the genetic background on the progression of liver diseases remains debatable. Several mutations associated with a high risk of HCC were commonly found in HIV-HBV coinfected patients, possibly explaining the high rates of carcinogenesis [64]. Nonetheless, double mutations in the core region (A1762T and G1764A), overlapping with the oncogenic HBx protein-encoding region, that were associated with advanced liver disease and HCC in HBV mono-infected patients, are less common in HIV coinfected ones [65,66].

ART treatment. Historically, in HIV mono-infected patients, the first-generation nucleoside analogs reverse transcriptase inhibitors (NRTI)—stavudine (d4T) and didanosine (ddI) carried a risk of severe microsteatosis and lactic acidosis [67]. The older non-nucleoside reverse transcriptase inhibitors (NNRTI)—nevirapine and efavirenz, as well as several protease inhibitors, were also associated with severe liver injury. However, none of these antiretrovirals are currently used for HIV-HBV coinfected individuals. The recommended dual-acting regimens are associated with a favorable effect on liver disease [68], without liver toxicity over 10 years, although there have been signals of a potential increase in end-stage liver diseases and hepatocellular carcinoma in the D:A:D study associated with cumulative exposure to several drugs, including tenofovir [69]. Mitochondrial toxicity and lactic acidosis remain rare events, even though drug-induced hepatotoxicity is more frequently reported than in mono-infected patients, especially during severe immunosuppression [55]. An early initiation of antivirals, as recommended by the START trial, can reduce the incidence of serious non-AIDS events, including decompensated liver disease [70].

## 3. Virological Monitoring of Therapeutic Success in HIV-HBV Coinfection

**Figure 2:**
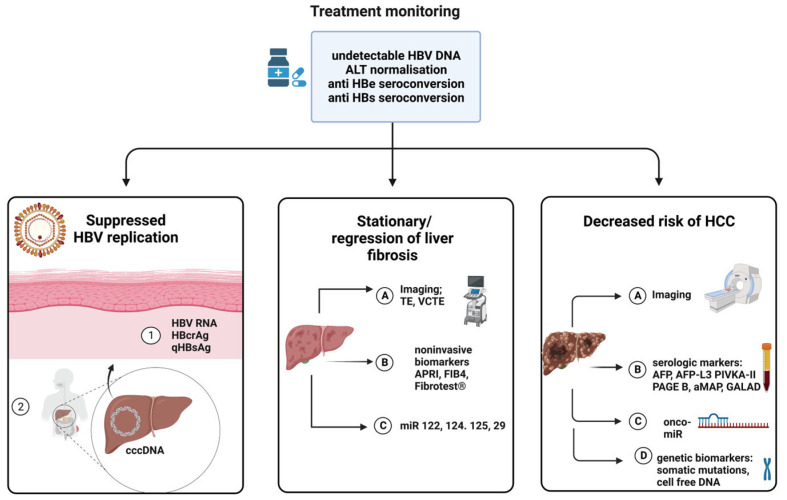
Biomarkers for treatment monitoring in HBV mono-infected and HIV-HBV coinfected patients. Three categories of biomarkers are useful for diagnosis and therapeutic follow-up: predictors of treatment efficacy (biomarkers used to assess viral replication), Biomarkers used to evaluate the degree of liver fibrosis, and those used to predict the risk of hepatocellular carcinoma (HCC). HBcrAg- hepatitis B core–related antigen, qHBsAg- quantitative hepatitis B surface antigen, cccDNA- covalently closed circular DNA, miR—microRNA, APRI-AST to Platelet Ratio, FIB4-Index combining age, AST, ALT and PLT count levels, AFP-alpha-fetoprotein, AFP-L3-*Lens culinaris* agglutinin-reactive fraction of alpha-fetoprotein; PIVKA-II-Protein induced by vitamin K absence or antagonist-II, PAGE-B—index comprising platelets, age, sex, and HBV infection, aMAP-index comprising age, gender, albumin, bilirubin and PLT count levels, GALAD—model including gender, age, AFP, L3-AFP and Des-carboxy-prothrombin, onco-miR—oncogenic microRNA. Created with BioRender.com (accessed on 26 April 2023), with a license to publish.

Four classic biomarkers are currently used to define therapeutic success in chronic hepatitis B in both immunocompetent and immunosuppressed patients: (1) HBV-DNA suppression (usually less than 60–80 IU/mL, although the lower quantification limit may differ among clinical trials), (2) ALT normalization, (3) anti-HBe-seroconversion and (4) HBsAg loss [22]. In addition, a systematic evaluation of liver fibrosis (every 3 months during the first year after diagnosis and every 6–12 months thereafter) using noninvasive markers and/or liver biopsy and hepatic ultrasound results are recommended for both HBV mono-infected and HIV-coinfected patients [21]. Nevertheless, virological and serological factors associated with long-term HIV-HBV coinfection evolution patterns are insufficiently understood, and, as a consequence, there is a continuous need for patient monitoring and testing. A series of novel biomarkers are now tested in HIV-HBV patients for an accurate diagnosis of viral replication, prediction of liver fibrosis, and HCC development. We present a synthetic view of the utility of these biomarkers for treatment monitoring and patients’ stratification according to the potential for severe evolution (Figure 2).

### 3.1. Predictors of Treatment Efficacy—Biomarkers of Viral Replication

#### 3.1.1. Covalently Closed Circular DNA (cccDNA)

cccDNA represents both the transcriptional template and the reservoir of HBV. Its lifelong persistence in an episomal form in the infected hepatocytes [71] and its potential for reactivation during immunosuppression, even in patients with presumed resolution of HBV infection, is the main obstacle for a complete cure of chronic hepatitis B. Monitoring the cccDNA level with ultrasensitive molecular biology techniques (Table 1) constitutes a promising biomarker for both mono- and coinfected patients, as a declining level has been associated with therapeutic success [71] and the probability of a functional cure. In HIV-HBV coinfected people with prolonged courses of dually active antiretrovirals, the transcription of cccDNA is diminished. The majority of these patients have undetectable levels of intra-hepatic cccDNA when assessed with the qPCR technique [72]. However, it is still detectable with droplet digital (ddPCR) assays [73] that allow the partition of PCR reactions into water-in-oil droplets and quantification of nucleic acid without a reference standard, with important advantages in terms of sensitivity, absolute quantification, and accuracy [74].

#### 3.1.2. Serum HBV-RNA

Serum HBV-RNA (also reported as preC RNA, pgRNA, or 3.5 kb RNA) can be detected as pre-genomic HBV-RNA and total serum HBV-RNA (for variants that cause defects in RNA splicing). Pre-genomic RNA indicates transcription of cccDNA and can be used in HBV mono-infected patients to identify cases (especially those with cirrhosis) who may safely discontinue HBV therapy or as a marker for the effectiveness of therapeutic agents that target cccDNA [75]. Data on the therapeutical implications of HBV-RNA detection in coinfected patients remains limited and requires an ongoing follow-up due to multiple fluctuations, irrespective of the baseline level, even on ART [76,77]. In HBV mono-infected patients, strong correlations were reported between HBV-RNA and both HBV-DNA and HBcrAg levels, while in HBV-HIV coinfected patients, the statistical correlations between various markers of HBV replication are less powerful [78,79]. Of note, in HIV-HBV coinfected patients, irrespective of their treatment status, pgRNA and total HBV-RNA are not associated with CD4 T cell count or HIV RNA [78,79].

#### 3.1.3. Hepatitis B Core–Related Antigen (HBcrAg)

HBcrAg contains three products encoded by the precore/core gene, which share an identical 149 amino acid sequence [80]. HBeAg—the soluble peptide derived from the precore protein by proteolysis; HbcAg—the viral nucleocapsid that remains in the hepatocyte; and p22 cr—a 22-kDa precore protein present in non-infectious HBV DNA-negative particles [81,82]. HBcrAg might be used in mono- and coinfected patients to identify those (a) who can safely discontinue nucleoside analogues therapy; (b) at risk for HBV reactivation during therapy; (c) at risk for carcinogenesis. A decrease in the HBcrAg level can be associated with undetectable levels of intra-hepatic cccDNA, followed by HBsAg loss—a major goal for a functional cure in chronic hepatitis B infection [80]. In HBV mono-infected patients, the association of quantitative HBcrAg with antiHBc antibody titers is used as a predictor of the level of intrahepatic cccDNA [69], especially during immune-modulatory therapies [44,73,74]. In HIV-HBV coinfected patients undergoing tenofovir therapy, the same combination can be used to monitor the kinetics and clearance of HBeAg [75].

#### 3.1.4. Quantitative HBsAg

The quantitative determination of HBsAg was correlated with intrahepatic replication. The level of HBsAg (expressed in IU/mL) is a widely available assay currently used to predict sustained virological response and achieve a functional cure for hepatitis B [82]. In HBV mono-infected patients, its clinical utility was mainly correlated with peginterferon treatment monitoring and liver fibrosis evaluation. In HbeAg-positive patients, an HBsAg level < 1000 IU/mL combined with an HBV-DNA level < 2000 IU/mL indicates minimal current viral activity [83]. Conversely, in spontaneous HBeAg seroconverters, with HBV-DNA level < 2000 IU/mL, an HBsAg level higher than 1000 IU/mL is associated with an increased risk of HBeAg-negative hepatitis, with active viral replication [83].

However, in treatment-naïve HBe positive patients, lower levels of HBsAg have been associated with higher levels of liver fibrosis [84] and are considered an indicator of an increased duration of liver disease and immune-mediated clearance of infected cells. These findings were not confirmed in Asian patients [84,85].

Recently, genotype-specific cutoffs of the HBsAg level have been proposed to rule out cirrhosis in HBeAg-positive patients [86]. The utility of qHBsAg testing for HIV-HBV coinfected patients is currently unclear.

### 3.2. Predictors of Liver Fibrosis

In efficiently treated HBV mono-infected patients, liver fibrosis remains stationary, and a certain degree of regression can occur in time. Information about the on-treatment evolution of liver fibrosis in HIV-HBV patients is incomplete and sometimes discordant. During the last decade, the role of liver biopsy became less prominent, as there was a continuous development of noninvasive tests that can accurately detect advanced stages of liver fibrosis and cirrhosis and predict liver-related outcomes of HIV-HBV infections.

#### 3.2.1. Fibrosis Indexes Based on Serum Biomarkers

APRI and FIB-4 scores, which utilize routine laboratory parameters, are very convenient and suitable noninvasive scores for liver fibrosis [118,119]. According to the most recent European Association for the Study of the Liver (EASL) recommendations, the regular calculation of simple, noninvasive fibrosis scores (FIB-4 or APRI) in populations at risk of liver fibrosis is recommended in combination with transient elastography (TE) for the stratification and linkage to care infections [120]; worth mentioning that neither TE nor noninvasive scores alone are considered appropriate to diagnose or exclude significant fibrosis.

FIB-4 score is based on a combination of age, AST, ALT, and PLT count levels and was first used for HIV-HCV coinfected patients. A lower limit of less than 1.45 indicates the absence of advanced fibrosis detected by liver biopsy (negative predictive value of 90%; sensitivity of 70%), while an upper limit higher than 3.25 indicates advanced fibrosis (with a positive predictive value of 65% and specificity of 97%) [121]. In subsequent studies, FIB-4 demonstrated a relatively high diagnostic value in patients with chronic hepatitis B when the threshold for advanced fibrosis was set at more than 2.0 (sensitivity of 69% and specificity of 95%) [122].

APRI index (AST to Platelet Ratio Index) is another noninvasive, inexpensive, and affordable score, tested initially on HCV-infected patients to assess the level of liver fibrosis; an APRI threshold of 0.7 was sensitive and specific for detection of significant fibrosis detected by liver biopsy (sensitivity of 77% and specificity of 72%, respectively), while for cirrhosis detection, an APRI cutoff of 1.0 was associated with 76% sensitivity and 72% specificity [123,124]. Subsequent studies have shown a good accuracy of the APRI score for HBV-induced cirrhosis [125,126].

Recent studies have shown that these scores are reliable noninvasive methods for the assessment of nonsignificant versus significant liver fibrosis in HIV-HBV coinfected patients also [127,128]. A FIB-4 value < 1.5 or APRI < 0.5 are concordant with Fibroscan results and can exclude fibrosis in 94.4% and 96.8% of cases [96].

Fibrometer score combines several markers (age, platelet count, prothrombin index, aspartate aminotransferase, alpha2-macroglobulin, hyaluronate, urea). The score’s sensitivity and specificity in predicting the advanced stages of fibrosis in patients with viral hepatitis are reported to be higher than those of the APRI test [129]. In HIV-HBV coinfected patients, the score is used as a noninvasive marker for the outcome of liver fibrosis after antiviral therapy [23].

Supplementary indexes, based on different inflammatory and anti-inflammatory cytokines, are now tested for HIV-HBV infection, as serum interleukins and interferon-gamma inducible protein-10 have been previously found dysregulated in both hepatitis B, C, HIV, or HIV-HCV coinfection [130,131,132], playing an important role in chronic hepatic inflammation and fibrogenesis.

#### 3.2.2. Imaging-Based Techniques for Detection of Liver Fibrosis

Recent studies using noninvasive tests for liver fibrosis assessment show that approximately 20–30% of HIV-HBV patients on long-term ART present with advanced liver fibrosis, with concordant results despite the variation of cutoffs. Data concerning advanced liver disease in HBsAg-positive PLH is further supported by studies using serological biomarkers and liver biopsies; the percentage of treatment-experienced patients displaying significant liver fibrosis in various studies can be seen in Table 2 [24,133].

Transient elastography (TE), an easily-performed and rapid test, is the most validated imaging-based technique in HBV infection [98]. It gives rapid information for clinical use, with a sensitivity of approximately 78% and a specificity of 81–82% for predicting significant fibrosis in HBV-infected patients, compared to liver biopsy [137,138]. TE has been increasingly used to document the stage of liver fibrosis in HIV-HBV coinfected patients. Studies on its accuracy have shown a good concordance with liver biopsy [100].

VCTE (Vibration-controlled transient elastography) predicts liver fibrosis accurately in patients with chronic hepatitis, irrespective of the etiology. In a large meta-analysis, VCTE showed good diagnostic performance in predicting significant fibrosis in patients with chronic hepatitis B, compared to liver biopsy (sensitivity between 71.2 and 99%, and specificity between 73.9 and 94% in different studies) [101].

These noninvasive methods can be used in HIV-HBV coinfected patients for longitudinal monitoring at shorter intervals. Nevertheless, limited data are available on the optimal cutoff in both naive and treated patients. Combining TE or VCTE with other serological markers might increase their diagnosis accuracy [120].

Serum markers and imaging-based techniques have both advantages over liver biopsy (including low risks, affordable cost, and better acceptance) and several disadvantages (including lower diagnostic accuracy for the prediction of early stages of fibrosis and inconsistent results in the presence of other comorbidities that can induce liver inflammation or fibrosis).

ART is generally associated with improving liver stiffness or improved liver fibrosis scores on liver biopsies, yet a subset of patients can display the progression of liver fibrosis [24]. Although sometimes this can be explained by suboptimal treatment adherence, there are patients with undetectable HIV-RNA levels who continue to display a low-level HBV viremia [139], often without persistent elevation of liver enzymes. In these cases, other conditions, including immune activation and additive metabolic factors [132], can play a role in the evolution of liver disease and viral replication [140]. The management of these patients is particularly challenging, and specific intervention must be directed to lifestyle modifications (a balanced diet, increased physical activity, elimination of additional risk factors such as alcohol intake and drug usage).

#### 3.2.3. MicroRNAs, as Potential Surrogate Biomarkers for Liver Fibrosis

Several microRNA species are constantly dysregulated during both HIV infection and chronic hepatitis B: miR-122, miR-210, and miR-181 expressions are increased, while miR-29 and miR-125 expressions are decreased [106]. The expression and the activity of these short transcripts in HBsAg-positive PLH are not very well understood, and their potential use as surrogate biomarkers for liver fibrosis is worth to be studied [141].

miR-122, a therapeutic target for HCV infection, is also directly correlated with increased necroinflammatory activity and advanced liver fibrosis [103] in HBV mono-infection. The serum levels of miR-122 are over-expressed in ART-treated HIV-1 patients before the development of liver disease [104], suggesting its potential clinical utility as a biomarker of severe liver disease evolution in coinfected patients.

miR-125, miR-124. The serum level of miRNA-125b is down-regulated and correlates with HBV viral load and is used either alone or combined with miRNA-124. It has the potential to discriminate between different grades of liver necroinflammation [142].

miR-29. Even though in HBV infection this transcript is reported to be downregulated, in treatment-experienced coinfected patients, miR-29 is upregulated, and associated with the virological and immunological markers of HIV-1 infection [143]. This is probably due to its involvement in the control of viral replication and immune responses [107].

The development of prediction scores, combining different miRNAs, can be an interesting approach to define the outcome of the HIV-HBV coinfected patients.

### 3.3. Tumor Markers and Predictors of Oncogenesis

#### 3.3.1. Classic Tumor Markers

The incidence of HCC is rising worldwide, being one of the most important causes of cancer death globally. In patients with chronic hepatitis B, antivirals that maintain HBV suppression can prevent or delay the evolution towards cirrhosis and HCC, but monitoring using abdominal ultrasound is still recommended in high-risk populations.

Several tumor biomarkers have been proposed, but generally failed in the early detection of HCC [117].

AFP (alpha-fetoprotein) is the classic marker used for HCC risk monitoring. According to the American Association for Study of Liver Diseases (AASLD) guidelines, an AFP level higher than 20 ng/mL indicates the need for follow-up [144,145], but due to its low diagnostic accuracy, it must be used in combination with other markers for HCC surveillance. An alternative biomarker-AFP-L3 (*Lens culinaris* agglutinin-reactive fraction of alpha-fetoprotein or highly sensitive -hs-AFP-L3) [146] can be tested using an increased sensitivity immunoassay, alone or in association with PIVKA-II (Protein induced by vitamin K absence or antagonist-II). The levels of these two proteins are usually increasing as HCC develops and progresses to portal vein invasion [108]. According to EASL guidelines, AFP-L3 is suboptimal in terms of cost-effectiveness for routine surveillance of early HCC, while PIVKA-II use has not been standardized until now [117].

Other biomarkers, such as Mac-2-binding protein glycosylation isomer (M2BPGi), a simple and reliable indicator of liver fibrosis in HBV mono-infection, used for prediction of hepatocarcinogenesis in patients treated with nucleoside analogs [109,110], have not yet been evaluated in HIV-HBV coinfection.

#### 3.3.2. Indexes Based on Biochemical Markers as Predictors of Oncogenesis

Different statistical models can generate indexes with higher specificity and predictive value for HCC evaluation and monitoring.

A recent score-*aMAP* (combining age, gender, albumin, bilirubin, and PLT count levels) was developed to assess the risk for carcinogenesis in patients with chronic hepatitis B and C, with a cutoff of 50 associated with a negative predictive value of 99.3–100% (sensitivity of 85.7–100%), irrespective of etiology [115].

PAGE-B index (comprising platelets, age, and gender) can predict the 5-year HCC risk in Caucasian HBV-infected patients with or without NA treatment [116,120]. EASL guidelines suggests that patients with a low PAGE-B score might even not require HCC surveillance [117].

GALAD score (calculated using gender, age, AFP, L3-AFP, and Des-carboxy-prothrombin) is a statistical model used for the early prediction of HCC development in patients with chronic hepatitis B. The association of the GALAD score with imaging-based techniques can generate better information for high-risk patient monitoring [111].

Still, none of these scores have been validated in HIV-HBV coinfected patients [147].

#### 3.3.3. Genetic Biomarkers

As appropriate biomarkers for early detection of HCC remain scarce, the addition of genetic biomarkers, such as somatic mutations in Telomerase Reverse Transcriptase (TERT) gene can further improve the diagnosis. Using liquid biopsy, several TERT promoter mutations (C228T and C250T) are frequently detected both in cirrhosis and HCC [148], and can be used to stratify patients with premalignant status for continuous monitoring.

Cell-free DNA (cfDNA—the nucleic acid released by cancerous cells that can be detected in peripheral blood) has occurred as a novel predictive biomarker for HCC. The methylation status of several genes, such as HOXA1, TSPLY5, and PFKP from cfDNA, has a good predictive value for hepatic oncogenesis [149,150].

### 3.4. Viral and Host miRNAs That Can Act as Oncogenes

HBV-encoded miRNAs. HBV-miR-2 is preserved among different HBV subtypes [106] and can act as an oncogene, promoting the development of HBV-related HCC and tumoral cell growth by suppressing apoptosis and stimulating migration and invasion [112]. HBV-miR-3, highly expressed in HBV chronic infection, is also considered an emerging factor in tumorigenesis, as it silences PPM1A (Protein phosphatase 1A) and promotes tumoral cell proliferation [113].

MiR-181, a cellular miRNA that has important roles in cell growth promotion, tumorigenesis, and apoptosis inhibition [114], is an epigenetic target for the HBV HBx gene. Its expression is upregulated in both HBV mono-infected and HIV-HBV coinfected patients, correlated with carcinogenesis [107].

New biomarkers, such as composite indexes of microRNAs, can help to better understand and predict the progression to liver fibrosis/cirrhosis and/or hepatocellular carcinoma. Such additional biomarkers have been proposed for the long-term follow-up of HBV mono-infected patients. Their relevance for the HIV-HBV coinfection and their value for routine clinical practice must be attentively appraised.

## 4. Conclusions

HIV-HBV coinfection remains a major public health problem. High rates of liver fibrosis, liver-related morbidity, hospitalizations, and mortality due to hepatocellular carcinoma represent important clinical challenges, especially in vulnerable, sometimes neglected populations. The combination between HIV and HBV replication, immune dysregulations, and immune-mediated hepatocellular injuries led to a more severe evolution compared to each of the mono-infection. Although the early initiation of antiretroviral therapy has an undeniably favorable impact on the evolution of HIV-HBV coinfection, access to highly potent dually active antiretrovirals remains imbalanced between different geographic regions. When therapy is delayed, the chronic immune activation and profound immunosuppression linked to HIV infection progression towards AIDS, with a reduced number of interferon-gamma-producing HBV-specific CD8+ T cells, can increase the risk of severe evolution [151,152].

Therefore, long-term monitoring of the active viral replication, the size and activity of the viral reservoir, and the progression of liver fibrosis using affordable and accurate tests is essential in order to prevent end-stage liver disease.

New noninvasive biomarkers reviewed in this manuscript are clinically relevant for HIV-HBV coinfected patients’ stratification, according to their risk for severe evolution and their response to therapy.

## Figures and Tables

**Table 1:** New biomarkers in chronic HBV mono-infection and HIV-HBV coinfection.

Biomarker	Diagnostic Test	Significance in Chronic HBV Mono-Infection	Significance in HIV-HBV Coinfection
1. Predictors of treatment efficacy			
cccDNA	q Rt PCR ddPCR RCA in liver biopsies	Viral reservoir, declining levels associated with therapeutic success [71]	Reduced transcription under prolonged ART [73]
Serum HBV-RNA	qRT-PCR RT-ddPCR [74]	Good correlation with cccDNA [75] indicates patients with cirrhosis who may safely discontinue HBV therapy.	Correlated with detectable HBeAg [76,77,78,79] and quantitative HBsAg [66,78,79] potential use to guide ART changes
HBcrAg	Chemiluminescence/EIA	Predict the evolution of liver disease and the risk of carcinogenesis [80,81]	Monitors the evolution and the clearance of HBeAg [82]
qHBsAg	EIA Chemiluminescence	Decreased in patients receiving treatment with nucleoside analogs and peginterferon therapy [83,84,85,86,87,88,89]	Decreased with an increasing number of CD4+ T cells [90,91,92]
2. Predictors of liver fibrosis			
Fibrosis indexes based on serum markers			
Fib-4 score	Index that combines age, AST, ALT, and PLT count levels	Diagnosis and follow-up of liver fibrosis (≥F2); can indicate the need to start HBV treatment; potential use as predictors of mortality [93,94,95]	Indicated for the screening of liver fibrosis in cases with elevated transaminases [94]
APRI	AST to Platelet Ratio	APRI threshold of 0.7 is sensitive and specific for the detection of significant fibrosis	An APRI < 0.5 are reported accordant with Fibroscan results to exclude fibrosis in 96.8% [96]
Fibrotest^®^	GGT, bilirubin, haptoglobin, α2-macroglobulin, apolipoprotein A1	Diagnosis, staging, and follow-up of liver fibrosis [94]	Good accuracy for the diagnosis of liver fibrosis [97]
Imaging-based techniques for the detection of liver fibrosis			
Transient elastography (TE)	FibroScan/Echosens, (Paris, France)	Good accuracy for the diagnosis staging and follow-up of liver fibrosis, including cases with normal/discrete ALT elevations [97,98,99]	Indicated in the diagnosis, staging, and follow-up of liver fibrosis in HIV-HBV coinfected patients [99,100].
VCTE	Vibration-controlled transient elastography	Predicts liver fibrosis accurately in patients with chronic hepatitis, irrespective of the etiology [101]	Noninvasive tool to assess liver fibrosis [32,52]
MicroRNAs as potential surrogate biomarkers for liver fibrosis			
miR-122	RT-PCR, 2^−ΔΔCT^ method [102]	Upregulated. Associated with the necroinflammatory activity, stage of fibrosis, HBsAg, and HBV DNA [103]	Biomarker of severe liver disease evolution in HIV infection [104]; more data needed for HIV-HBV coinfection
miR-125	RT-PCR; 2^−ΔΔCT^ method	Downregulated, correlates with HBV viral load and necroinflammatory activity	In HIV infection negatively correlate with HIV-RNA [105]; more data is needed for HIV-HBV coinfection
miR-29	RT-PCR; 2^−ΔΔCT^ method	Downregulated in HBV infection [106]	Over-expressed in HIV infection, control of viral replication (mechanism unknown) [107]
3. Tumor markers			
Classic tumor markers			
AFP/PIVKA-II/AFP-L3	EIA IHC	Usually increase as HCC develops, irrespective of the etiology	No data [108]
M2BPGi	Lectin-antibody sandwich immunoassay	Marker for liver fibrosis stage	No data [109,110]
GALAD score	Statistical model that includes gender, age, AFP, L3-AFP, and Des-carboxy-prothrombin (DCP)	Predicts early HCC The association with ultrasound and elastography increases the performance	No data [111]
miRNAs as oncogenes			
HBV-miR-2	Deep sequencing technology	HBV-encoded miRNA; promotes tumoral cell growth by suppressing apoptosis	No data [112]
HBV-miR-3	Deep sequencing technology	HBV-encoded miRNA; promotes tumoral cell growth by silencing PPM1A (Protein phosphatase 1A)	No data [113]
MiR-181	RT-PCR; 2^−ΔΔCT^ method	Cellular miRNA; promotes cell growth, tumorigenesis, and decreasing of apoptosis; it is an epigenetic target for the HBX gene of HBV [114]	Similar activity in HIV-HBV coinfected patients correlated with carcinogenesis [107]
Indexes based on biochemical markers as predictors of oncogenesis			
aMAP	Index that comprises age, gender, albumin, bilirubin, and PLT count levels	A cutoff value of 50 is predictive of the risk of carcinogenesis [115]	Not yet tested
PAGE-B index	Index that comprises platelets, age, sex, and HBV infection	Carcinogenesis prediction in treated HBV-infected patients [116] Recommended by EASL guidelines to delay the HCC surveillance [117]	Not yet tested

Legend: HBcrAg—hepatitis B core-related antigen, qHBsAg—quantitative hepatitis B surface antigen, AFP—alpha-fetoprotein, PIVKA-II—protein induced by vitamin K absence or antagonist-II, AFP-L3-*Lens culinaris* agglutinin-reactive fraction of alpha-fetoprotein; M2BPGi—Mac-2-binding protein glycosylation isomer; qRT PCR—quantitative real-time PCR; ddPCR—digital droplet-polymerase chain reaction; RCA—rolling circle amplification; RT-ddPCR—reverse-transcription ddPCR; EIA—enzyme immunoassay, IHC—immunohistochemical analysis, LiPA—line probe assay; EASL—European Association for the Study of the Liver; HCC—hepatocellular carcinoma.

**Table 2:** Prevalence of liver fibrosis in HIV-HBV infected patients on ART.

Author	Study Group	Assessment Method	Median ART Duration (Years)	Median CD4 T Cell Count	Undetectable HIV-RNA/Undetectable HBV DNA (%)	Prevalence of Liver Fibrosis
Iacob, D., 2022 [127]	212 HIV infected, 101 HIV-HBV coinfected	APRI and Fib-4 scores	13	369	68%/46%	10.8% at baseline, 11.3% at 5 years follow up
Sterling, R.K., 2018 [133]	114 HIV-HBV patients	Liver Histology, APRI and Fib-4 scores	14	568	77.9%/57.9%	37% significant fibrosis (Ishak ≥ 2) 24% advanced fibrosis (Ishak ≥ 3)
Maida, I., 2006 [134]	37 HIV-HBV patients	Transient elastography	3.3	490	89%/70%	57% no or mild fibrosis 13% significant fibrosis (F3) 11% advanced fibrosis (F4)
Audsley, J., 2016 [135]	70 HIV-HBV patients, of which 20 co-infected with HCV	Transient elastography	10	381	74.6%/74.6%	35.7% significant fibrosis (≥F3)
Boyd, A., 2017 [24]	148 HIV-HBV patients, of which 12 co-infected with HDV 19 co-infected with HCV	Fibrometer score and liver biopsy	5.7	420	53.4%/17.8%	31% F3–F4 fibrosis
Miailhes, P., 2011 [136]	59 patients (46 patients on cART)	Transient elastography, liver biopsy, and Fibrotest	Not specified	397	85%/78%	33.8% F3–F4 fibrosis
Stockdale, A.J., 2015 [98]	106 HIV-HBV patients on ART and 15 ART-naive	Transient elastography	3.75	571	67%/49.1%	2.7% F3–F4 fibrosis

## Data Availability

Not applicable.
